# Enterovirus A71 Infection and Neurologic Disease, Madrid, Spain, 2016

**DOI:** 10.3201/eid2501.181089

**Published:** 2019-01

**Authors:** Carmen Niño Taravilla, Isabel Pérez-Sebastián, Alberto García Salido, Claudia Varela Serrano, Verónica Cantarín Extremera, Anna Duat Rodríguez, Laura López Marín, Mercedes Alonso Sanz, Olga María Suárez Traba, Ana Serrano González

**Affiliations:** Hospital Infantil Universitario Niño Jesús, Madrid, Spain

**Keywords:** Enterovirus, epidemic, epidemiology, meningitis/encephalitis, encephalomyelitis, viruses, Madrid, Spain, neurologic disease

## Abstract

We conducted an observational study from January 2016 through January 2017 of patients admitted to a reference pediatric hospital in Madrid, Spain, for neurologic symptoms and enterovirus infection. Among the 30 patients, the most common signs and symptoms were fever, lethargy, myoclonic jerks, and ataxia. Real-time PCR detected enterovirus in the cerebrospinal fluid of 8 patients, nasopharyngeal aspirate in 17, and anal swab samples of 5. The enterovirus was genotyped for 25 of 30 patients; enterovirus A71 was the most common serotype (21/25) and the only serotype detected in patients with brainstem encephalitis or encephalomyelitis. Treatment was intravenous immunoglobulins for 21 patients and corticosteroids for 17. Admission to the pediatric intensive care unit was required for 14 patients. All patients survived. At admission, among patients with the most severe disease, leukocytes were elevated. For children with brainstem encephalitis or encephalomyelitis, clinicians should look for enterovirus and not limit testing to cerebrospinal fluid.

## Abstract 2

For children with brainstem encephalitis or encephalomyelitis, clinicians should look for enterovirus and not limit testing to cerebrospinal fluid.

Enteroviruses (family *Picornaviridae*) are RNA viruses; the >100 recognized enterovirus types are classified into 4 species, A–D. Enterovirus infections are common worldwide and occur mostly among children; infections are usually asymptomatic or mild but can produce severe neurologic disease (*1*). Enterovirus A71 (EV-A71) has emerged as an etiologic agent of aseptic meningitis, encephalitis, encephalomyelitis, acute flaccid paralysis, and other severe systemic disorders, including neurogenic pulmonary edema, and cardiopulmonary failure (*2*). The first outbreak of EV-A71–associated neurologic disease occurred in California in the 1960s (*3*). Since then, epidemics have been reported in the Asia-Pacific region (*4*–*7*). In Europe, an outbreak was first described in Bulgaria and Hungary in the 1970s (*8*,*9*). During 2016, an outbreak of enterovirus with neurologic involvement was detected in Spain. The first case described was that of acute flaccid paralysis associated with enterovirus-D68 in Catalonia, followed by another 3 cases in Aragón, Galicia, and Asturias. During April–June 2016 in Catalonia, several cases of brainstem encephalitis and encephalomyelitis associated with EV-A71 infection affected >100 children (*10*–*16*). Since May 2016, increased numbers of enterovirus central nervous system (CNS) infections have been observed in Madrid.

In this article, we describe the epidemiology, clinical data, therapies, and clinical progression of children hospitalized at Hospital Infantil Universitario Niño Jesús of Madrid during 2016 because of neurologic signs and symptoms and confirmed enterovirus infection. We also describe which variables were associated with severe disease and worst prognoses.

We enrolled only patients for whom written informed consent was obtained from the next of kin, caretakers, or guardians on behalf of the children. The study was approved by the Hospital Infantil Universitario Niño Jesús of Madrid (Spain) ethics committee. The research was conducted according to The Code of Ethics of the World Medical Association (Declaration of Helsinki).

## Material and Methods

### Study Design

This study was an observational prospective study of children admitted to the Hospital Infantil Universitario Niño Jesús of Madrid from January 2016 to January 2017 because of neurologic symptoms with suspicion of enterovirus infection (meningitis, meningoencephalitis, encephalomyelitis, brainstem encephalitis, or acute flaccid paralysis). The hospital is a 230-bed reference medical center for patients with high-complexity pathologic conditions.

We included children with a clinical diagnosis of meningitis, encephalitis, brainstem encephalitis, encephalomyelitis with or without autonomic dysfunction, or flaccid paralysis resulting from spinal cord involvement. We excluded children with neurologic impairment but no enterovirus isolation.

#### Data Collection and Diagnosis

For each patient, we collected demographic, clinical, biological, microbiological, and radiographic data. Demographic data were patient sex, age in months, and hospitalization duration. Clinical data were past medical condition(s); fever duration; respiratory, gastrointestinal, and neurologic symptoms; and mucocutaneous manifestations. Biological data were leukocytosis (>15,000 leukocytes/mm^3^), thrombocytosis (>400,000 thrombocytes/mm^3^), and C-reactive protein and procalcitonin levels. Microbiological data were blood and cerebrospinal fluid (CSF) analysis and bacterial culture results. For each patient, we tested CSF for enterovirus by real-time PCR and for herpes simplex virus by reverse transcription PCR. If those PCRs were negative, we collected nasopharyngeal aspirates and anal swab samples for enterovirus detection by real time-PCR. Enterovirus-positive samples were genotyped at the Enterovirus Unit of the National Centre for Microbiology (Institute of Public Health “Carlos III,” Madrid, Spain). Radiologic data were from imaging studies performed on all children with rhomboencephalitis, acute flaccid paralysis, or neurologic deterioration. Studies included brain and spine magnetic resonance imaging (MRI).

#### Management and Follow-Up

Patients with meningoencephalitis or rhomboencephalitis and substantial somnolence or incipient bulbar clinical signs received intravenous immunoglobulins (IVIG) (400 mg/kg 1×/d for 5 d). Patients with signs of bulbar or medullary involvement or with lesions suggestive of rhomboencephalitis seen on MR images received methylprednisolone (30 mg/kg 1×/d for 3–5 d) and fluoxetine (0.3 mg/kg 1×/d for 14 d). Each patient underwent a follow-up examination at 1 and 3 months after hospital admission. To stratify the severity of illness, we used the World Health Organization (WHO) Guide to Clinical Management and Public Health Response for Hand, Foot and Mouth Disease (*14*). The 6 clinical conditions classified by WHO are aseptic meningitis, encephalitis, brainstem encephalitis (rhomboencephalitis), encephalomyelitis, autonomic nervous system dysfunction, and cardiopulmonary failure.

### Statistical Analyses

We report descriptive statistics in terms of absolute frequencies and percentages. For data comparisons of categorical variables, we used Pearson χ^2^ or Fisher exact tests when appropriate. We describe continuous nonnormal distributed variables as median values and interquartile ranges (IQRs) and compared them by using the Mann-Whitney U test and Kruskal-Wallis analysis. Statistical analyses were performed with SPSS version 22.0 software (https://www.ibm.com/es-es/marketplace/spss-statistics). We considered p<0.05 as statistically significant.

## Results

### Epidemiologic Data

**Figure 1:**
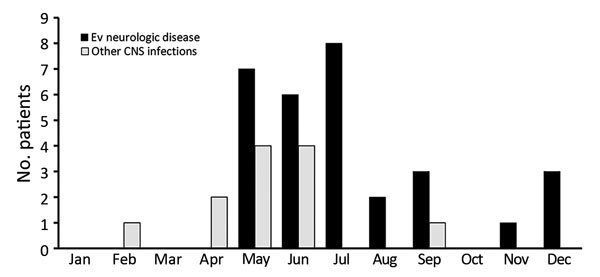
Monthly distribution of patients admitted to Hospital Infantil Universitario Niño Jesús, Madrid, Spain, for CNS infections in 2016. CNS, central nervous system; EV, enterovirus.

During the study period, 42 patients were hospitalized for suspected enterovirus neurologic disease. For 12 patients, other causes for their neurologic signs were identified or enterovirus was not isolated. Of the 30 remaining patients, median age was 23 months (IQR 16–41 months), 18 were male, and 26 became ill during May–September (peak incidence [8 cases] in July) (Table; Figure 1).

**Table:** Enterovirus serotype, localization of isolation, WHO clinical classification, and outcomes for 30 patients with enterovirus infection and neurologic disease, Madrid, 2016*

Patient no.	Patient age/sex	WHO clinical classification	Enterovirus source	Enterovirus serotype	Patient outcome
1	2 mo/M	Aseptic meningitis	CSF	Genotyped negative	Recovered
2	16 d/F	Aseptic meningitis	CSF	ND	Recovered
3	3 mo/F	Encephalitis	Nasopharyngeal aspirate	A71	Recovered
4	10 mo/F	Encephalitis	Nasopharyngeal aspirate	A71	Recovered
5	12 mo/M	Brainstem encephalitis	Nasopharyngeal aspirate	A71	Unknown
6	17 mo/F	Brainstem encephalitis	Nasopharyngeal aspirate	Genotyped negative	Unknown
7	21 mo/F	Brainstem encephalitis	Nasopharyngeal aspirate	A71	Cerebellar dysfunction
8	22 mo/F	Encephalitis	Nasopharyngeal aspirate	Rhinovirus	Recovered
9	19 mo/M	Encephalitis	Nasopharyngeal aspirate	A71	Unknown
10	18 mo/M	Encephalitis	Anal swab sample	A71	Cerebellar dysfunction
11	2 y/M	Brainstem encephalitis	Nasopharyngeal aspirate	A71	Recovered
12	2 y/M	Cardiopulmonary failure	Nasopharyngeal aspirate	A71	Acquired brain damage
13	23 mo/F	Cardiopulmonary failure	Anal swab sample	A71	Cerebellar dysfunction
14	2 y/M	Encephalitis	Nasopharyngeal aspirate	B	Recovered
15	3 y/M	Brainstem encephalitis	Nasopharyngeal aspirate	A71	Recovered
16	3 y/F	Brainstem encephalitis	Anal swab sample	A71	Recovered
17	3 y/F	Cardiopulmonary failure	Nasopharyngeal aspirate	A71	Paresis of the right upper limb
18	4 y/F	Brainstem encephalitis	Nasopharyngeal aspirate	A71	Cerebellar dysfunction
19	4 y/M	Aseptic meningitis	CSF	ND	Recovered
20	4 y/M	Brainstem encephalitis	Nasopharyngeal aspirate	A71	Cerebellar dysfunction
21	4 y/M	Aseptic meningitis	CSF	Echovirus	Recovered
22	6 y/M	Aseptic meningitis	CSF	ND	Recovered
23	6 y/F	Aseptic meningitis	CSF	Echovirus	Recovered
24	7 y/M	Brainstem encephalitis	Nasopharyngeal aspirate	A71	Cerebellar dysfunction
25	1 mo/M	Encephalitis	CSF	A71	Recovered
26	4 y/M	Encephalitis	Nasopharyngeal aspirate	A71	Recovered
27	5 y/M	ANS dysfunction	Nasopharyngeal aspirate	A71	Cerebellar dysfunction
28	2 y/F	Encephalitis	Nasopharyngeal aspirate	A71	Recovered
29	2 y/M	Brainstem encephalitis	Anal swab sample	A71	Recovered
30	20 mo/M	Brainstem encephalitis	Anal swab sample	A71	Peripheral facial paralysis

*ANS, autonomic nervous system; CSF, cerebrospinal fluid; ND, not done; WHO, World Health Organization.

### Clinical Manifestations

Of the 30 patients included in the study, fever (axillary temperature >38°C [100.4°F]) was observed for 22, vomiting for 21, fatigue for 20, headache for 11, catarrhal symptoms for 6, and diarrhea for 2. Mucocutaneous manifestations were observed for 6 patients; the main manifestation was petechial rash on the extremities. The most common neurologic sign among the 30 patients was lethargy or drowsiness for 24, followed by myoclonic jerks for 4, tremor for 7, and ataxia for 17. One patient had tetraparesis and another had paresis isolated to the right arm. Cardiorespiratory failure (cardiogenic shock and neurogenic pulmonary edema) developed in 3 patients; 1 experienced cardiac arrest, which was reversed with advanced cardiopulmonary resuscitation maneuvers. For those who experienced them, fever and neurologic signs started a median of 3 days (IQR 1.25–5 days) before hospitalization.

### Supplementary Testing

Blood analysis revealed leukocytosis in 10 patients and thrombocytosis in 9. No patient had significant alterations in plasma C-reactive protein levels, and only 1 showed elevated procalcitonin levels (3.6 ng/mL [reference value 0.5 ng/mL]).

All patients underwent lumbar puncture. CSF analyses showed a median leukocyte count of 112 leukocytes/mm^3^ (IQR 28–211 leukocytes/mm^3^) and an average mononuclear cell percentage of 56% (IQR 19.5%–90.0%). For only 3 patients CSF contained <10 leukocytes/mm^3^ (reference count). In the CSF, the median level of protein was 32.5 mg/dL (IQR 26–46 mg/dL) and of glucose 62.5 mg/dL (IQR 55–67 mg/dL). In no patient was the glucose level <50 mg/dL.

Real-time PCR detected enterovirus in CSF of 8 of the 30 patients, all of whom had aseptic meningitis. Patients for whom CSF analysis was negative underwent nasopharyngeal aspirate or anal swab sample testing for enterovirus by the same real-time PCR. An enterovirus was isolated from nasopharyngeal aspirate of 17 of the 30 patients and from anal swab samples of 5. The enterovirus from 25 of 30 patients was genotyped; most frequently identified was EV-A71 (21 patients), followed by echovirus (2 patients), enterovirus B (1 patient), and rhinovirus (1 patient). EV-A71 was the only serotype detected in patients with brainstem encephalitis or encephalomyelitis and was isolated from respiratory and fecal samples. Patients from whom other enteroviruses (echovirus, enterovirus B, or rhinovirus) were isolated received a diagnosis of meningitis or encephalitis. No cultures of CSF or blood were positive for bacteria. In no patient was herpes simplex virus detected in CSF.

**Figure 2:**
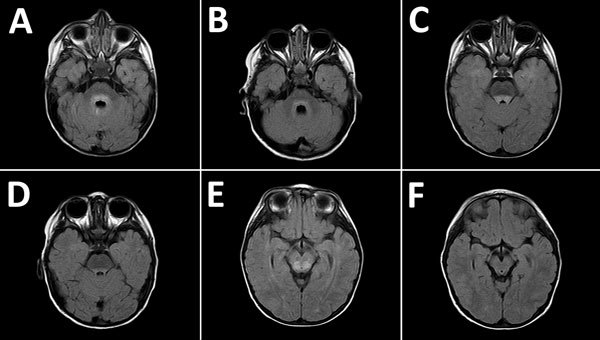
Magnetic resonance images of the brain of a 2-year-old boy with enterovirus meningoencephalitis. A–C) Brain at time of diagnosis. FLAIR sequences show hyperintense lesions around ventricle IV (A), posterior region of the pons (B), and posterior region of the mesencephalon (C). D–F) Control images of cerebrum 6 months after diagnosis. FLAIR sequences show slight hyperintensity of signal around ventricle IV, lower than in the initial study (D), and complete resolution of lesions in the posterior region of the pons (E) and mesencephalon (F).

**Figure 3:**
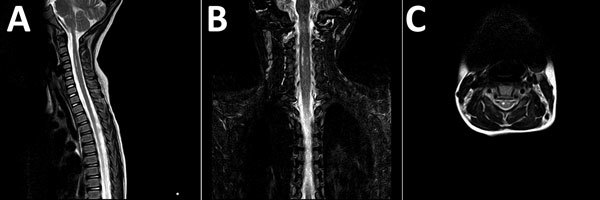
Magnetic resonance images of brain of a 3-year-old girl with enterovirus encephalomyelitis (paresis of the right upper limb). A) Image of the cervical spine: sagittal T2 sequence; B) short tau inversion recovery (STIR) coronal sequence; C) T2 axial sequence. Hyperintense filiform lesions in the anterolateral regions of the spinal cord (C3–C5), predominantly right, are suggestive of myelitis.

MRI was performed for 26 of the 30 patients; results were within normal limits for 5. Among the 26 patients, the radiologic abnormalities identified were leptomeningeal enhancement in 5, alteration of the signal of the white matter in the rhomboencephalic region (bulb, protuberance, cerebellum, or fourth ventricle) in 16, and cervical myelopathy in 3. (Figures 2, 3).

### Treatment

Of the 30 patients, 21 received IVIG; 17 received corticosteroids, and 11 of those 17 received corticosteroids and fluoxetine. No patient with aseptic meningitis or encephalitis received treatment.

### Outcomes and Variables Associated with More Severe Disease

Median hospital stay was 10 days (IQR 6–14.5 days). Fourteen patients were admitted to the pediatric intensive care unit (PICU) because of decreased consciousness level (9 patients), paresis (1 patient), or automatic nervous system dysregulation (4 patients, among whom 3 exhibited cardiorespiratory failure and required mechanical ventilation and treatment with an inotrope such as milrinone). The median PICU stay was 9.5 days (IQR 1.5–47 days). None of these patients died.

At the time of hospital discharge, 20 children experienced no sequelae, 7 had cerebellar dysfunction that consisted of slightly wide foot placement while walking or slight instability while sitting, 1 had paresis of the right upper limb, and 1 had peripheral facial paralysis. Another patient had acquired brain damage. Follow-up examination at 3 months after hospital admission detected only slight cerebellar alteration in 2 patients and a mild motor deficit with difficulty extending the right upper limb in 1 patient.

Of the patients with autonomic nervous system dysregulation and cardiorespiratory failure, 1 had acquired brain damage requiring a tracheostomy and a nasogastric tube for feeding. At the time of the most recent follow-up examination (June 30, 2018), the patient no longer required mechanical ventilation or the nasogastric tube for feeding.

In terms of clinical and analytical criteria, only the number of leukocytes in the blood at the time of admission was significantly higher in patients with the most severe disease (according to WHO classification) (p = 0.00). Of the 30 patients, all 3 with cardiopulmonary failure exhibited bulbar inflammatory lesions on MR images, but this finding was also found in 13 patients without autonomic nervous system dysfunction.

## Discussion

The outbreak we report occurred in spring and summer, the typical prevalence pattern of enterovirus in Spain (*17*). The first outbreak of CNS disease associated with EV-A71 in Spain occurred in March 2016, when the number of children with neurologic syndromes caused by enterovirus infection in Catalonia increased (*13*,*15*). In the rest of Spain, some sporadic cases occurred, but no reports were published. In our case series of concurrent neurologic disease and enterovirus infection, the main cause was EV-A71. In the Catalonia outbreak and in the cases reported here, infection with enterovirus D68, a serotype that may cause an acute flaccid polio-like paralysis in children with previous respiratory infections, was ruled out. Since 2012, the incidence of fever and hand, foot, and mouth disease (HFMD) caused by EV-A71 was increasing in Spain, but until 2016 this virus had not produced severe neurologic disease (*16*).

The clinical characteristics of the patients in our study were similar to those of patients described in other case series: young children (<5 years of age) with fever and respiratory or gastrointestinal symptoms, followed 3–5 days later by neurologic signs. The rate of mucocutaneous manifestations in patients in our study was low. The neurologic signs were drowsiness, ataxia, and positive meningeal signs, concordant with signs described in the literature (*12*,*13*,*18*,*19*). Myoclonic jerks were rare among patients in our study compared with patients in other case series (*20*,*21*).

Detection of EV-A71 from sterile sites is specific but usually insensitive. Virus is detected in 0%–5% of CSF samples from patients with neurologic disease. Real-time PCR for enterovirus in CSF was negative for 83.3% of patients in our study, and most diagnoses were made from nasopharyngeal aspirates or anal swab samples, as described elsewhere (*1*,*2*,*10*–*15*,*18*,*19*,*22*). This finding may be because the neurologic involvement is caused by an immune-mediated response (*22*,*23*) or because of earlier enterovirus elimination from CSF (*19*). Consequently, diagnostic assessments should include specimens from multiple sites. Disease was less severe for patients with positive PCR results for enterovirus in CSF.

Of the radiologic studies, the most specific was the MRI, which showed tegmental-protuberancial involvement (characteristic high-signal intensities on T2-weighted images). A restricted diffusion pattern was observed in images of patients with severe disease (the 3 patients with cardiopulmonary failure). In this study, lesions detected on MR images coincided with those described in the literature (*12**,**19**,**24**,**25*); we found a high number of patients who had nonsevere symptoms and MR images indicating bulbar involvement.

There is no proven effective therapy for EV-A71 infections. Antiviral drugs, corticosteroids, and IGIV have been used. The treatment given to patients in this series was chosen because it had been effective in other patients with enterovirus infection and CNS disease (*1*,*2*,*11*–*15*,*18*–*22*,*26*–*30*) and because it is a common treatment for viral and inflammatory myelitis (*31*). During the first outbreaks in Asia, IGIV was already in use, and a retrospective comparison suggests that this treatment was beneficial (*32*,*33*). However, the use of corticosteroids has been questioned after publication of a comparative study (*34*). In our experience, all patients with severe disease received corticosteroids, even though WHO does not recommend them for patients with HFMD of any severity.

Fluoxetine inhibits replication of enterovirus B and D but not of enterovirus A or C or of rhinovirus (*35*–*37*). At the time of admission, some of our patients received fluoxetine because we did not know the type of enterovirus; after the serotype was known to be A71, use of fluoxetine was not indicated.

Several antiviral drugs are available. Pleconaril is an antiviral drug that inhibits the entry of enteroviruses into cells, but it is not active against enterovirus serotypes A71 or D68 (*28*). Other antiviral drugs, such as pocapavir and vapendavir, have not been shown to be effective (*38*).

Concern about enterovirus outbreaks and the lack of effective treatment is growing. Many EV-A71 vaccines have been studied (*39*–*45*). In China, 2 inactivated EV-A71 vaccines have been approved for the prevention of severe HFMD (*46*). Efficacy, immunogenicity, and safety were reported, concluding that vaccine efficacy was 94.84% (CI 83.53%–98.38%) and, in the second year, 100% (CI 84.15%–100%) against HFMD related to EV-A71 (*47*). No serious adverse events related to the vaccine have been described (*47*). As a result of these data, the potential severity of disease, and the lack of etiologic treatment, this vaccination could be the most useful preventive strategy.

The severity of the neurologic involvement and the lethality of the different outbreaks described for enterovirus vary (*1*,*2*,*10*–*15*,*18*–*22*). In our case series, prognoses were favorable, and no patients died. However, 1 in 2 patients was admitted to the PICU. Three patients with autonomic nervous system dysfunction and cardiopulmonary failure required mechanical ventilation and inotropes. One required tracheostomy and feeding by nasogastric tube, and another experienced paresis of the right upper limb. We found statistically significant leukocytosis only in the most severely affected children, as has been described (*1*,*2*,*19*).

Our study has several limitations because it is an observational single-center study. The treatment used was empirically prescribed, thus limiting conclusions about effectiveness. The rate of sequelae was low, but long-term follow-up was not conducted. A multicenter study would be desirable.

In conclusion, the serotype most frequently isolated from the patients in our series was EV-A71. PCRs from respiratory and gastrointestinal tract samples had higher diagnostic value than PCRs of CSF. Treatment with IVIGs and corticosteroids was administered according to disease severity. Higher leukocyte counts were associated with more severe disease. There were no deaths and only 2 patients experienced significant sequelae, but almost half of the patients required admission to the PICU. For children with brainstem encephalitis or encephalomyelitis, infection with EV-A71 should be suspected. Diagnostic testing should include nasopharyngeal and anal swab samples as well as CNS fluid.
